# The vaccinia virus based Sementis Copenhagen Vector vaccine against Zika and chikungunya is immunogenic in non-human primates

**DOI:** 10.1038/s41541-020-0191-8

**Published:** 2020-06-02

**Authors:** Natalie A. Prow, Liang Liu, Mary K. McCarthy, Kevin Walters, Raj Kalkeri, Jillian Geiger, Fusataka Koide, Tamara H. Cooper, Preethi Eldi, Eri Nakayama, Kerrilyn R. Diener, Paul M. Howley, John D. Hayball, Thomas E. Morrison, Andreas Suhrbier

**Affiliations:** 1 Inflammation Biology, QIMR Berghofer Medical Research Institute, Brisbane, QLD 4029 Australia; 2 Australian Infectious Disease Research Centre, Brisbane, QLD 4029 and 4072 Australia; 3 Experimental Therapeutics Laboratory, School of Pharmacy and Medical Sciences, UniSA Cancer Research Institute, University of South Australia, Adelaide, SA 5000 Australia; 4 Department of Immunology and Microbiology, University of Colorado School of Medicine, Aurora, CO 80045 USA; 5 Department of Infectious Disease Research, Southern Research Institute, Frederick, MD 21701 USA; 6 Department of Virology I, National Institute of Infectious Diseases, Tokyo, 162-8640 Japan; 7 Robinson Research Institute and Adelaide Medical School, University of Adelaide, Adelaide, SA 5005 Australia; 8 Sementis Ltd, Berwick, VIC 3806 Australia

**Keywords:** Biotechnology, Immunology, Microbiology

## Abstract

The Sementis Copenhagen Vector (SCV) is a new vaccinia virus-derived, multiplication-defective, vaccine technology assessed herein in non-human primates. Indian rhesus macaques (*Macaca mulatta*) were vaccinated with a multi-pathogen recombinant SCV vaccine encoding the structural polyproteins of both Zika virus (ZIKV) and chikungunya virus (CHIKV). After one vaccination, neutralising antibody responses to ZIKV and four strains of CHIKV, representative of distinct viral genotypes, were generated. A second vaccination resulted in significant boosting of neutralising antibody responses to ZIKV and CHIKV. Following challenge with ZIKV, SCV-ZIKA/CHIK-vaccinated animals showed significant reductions in viremias compared with animals that had received a control SCV vaccine. Two SCV vaccinations also generated neutralising and IgG ELISA antibody responses to vaccinia virus. These results demonstrate effective induction of immunity in non-human primates by a recombinant SCV vaccine and illustrates the utility of SCV as a multi-disease vaccine platform capable of delivering multiple large immunogens.

## Introduction

Poxvirus-based vaccine vector systems have a number of attractive features including (i) the ability to accommodate large recombinant immunogen payloads (at least 25,000 base pairs), (ii) a capacity for cold chain-independent distribution, (iii) the lack of DNA integration and (iv) the potential for needle-free vaccine delivery (reviewed in ref. ^1^). A range of vaccine vector systems based on vaccinia virus (VACV) and other poxviruses have been developed, with several sold as products and many more in human clinical trials^1,2^. These include NYVAC^3^, ALVAC^4^, fowlpox^5^ and Modified Vaccinia Ankara (MVA)^6^, with a large series of recombinant MVA (rMVA) vaccines evaluated in non-human primate (NHP) studies^2,7^ and in human clinical trials^1,8^. In humans and NHPs, rMVA vaccines are generally effective at boosting immune responses, but are often poor as stand-alone vaccines for induction of effective immune responses to recombinant vaccine antigens in naive individuals. Heterologous prime-boost strategies (e.g., DNA prime and rMVA boost) have been widely adopted to overcome this limitation^1,7,9,10^. MVA was rendered replication defective in mammalian cells by 572 passages of VACV in primary chicken embryo fibroblasts (CEFs) and, towards the end of the global smallpox, vaccination campaign was used in ≈120,000 people with no significant side effects. MVA is currently sold as a smallpox vaccine as IMVANEX/IMVAMUNE (by Bavarian Nordic)^8,11^. Manufacture of rMVA/MVA vaccines has relied on the use of CEFs, although EB66, a duck embryo-derived cell line, may be used in the future^12^. The other aforementioned poxvirus vectors similarly use CEFs for production.

The Sementis Copenhagen Vector (SCV) represents a new vaccine vector technology based on the Copenhagen strain of VACV. SCV is unable to generate infectious viral progeny in vaccine recipients due to a targeted deletion of *D13L*, a gene encoding the essential viral assembly protein, D13. Recombinant SCV vaccines are produced in Chinese Hamster Ovary (CHO) cells modified to express D13 and the host range protein CP77. CHO cells are widely used in the manufacture of biologics and provide a significant advantage over CEFs that have traditionally been used for the manufacture of VACV-based vaccines^1,13,14^. A single-construct, multi-pathogen SCV vaccine encoding the structural gene cassettes of chikungunya virus (CHIKV) and Zika virus (ZIKV) (SCV-ZIKA/CHIK) was recently shown to be immunogenic and protective against both viruses in a series of mouse challenge models after a single vaccination^14^. An SCV vaccine was also shown to protect against ectromelia virus (a mouse model of smallpox) and to be non-pathogenic in SCID mice (a mouse model of lethal progressive VACV infection)^13^.

The largest outbreak of CHIKV ever recorded started in 2004 and reached >100 countries in four continents, with >10 million cases of primarily rheumatic disease, with mortality rate estimates ranging from 0.024 to 0.7% of CHIKV cases^15^. In 2016, the World Health Organization declared the ZIKV pandemic a public health emergency of international concern. ZIKV is the aetiological agent of Congenital Zika Syndrome (CZS), a spectrum of primarily neurological abnormalities (including microcephaly) in newborns arising from ZIKV infection of pregnant mothers, with >4300 CZS cases reported in Brazil in 2016^16^. A considerable international effort has been underway to develop vaccines against both CHIKV^17^ and ZIKV^18^. A combination ZIKV and CHIKV vaccine is deemed attractive as these two arboviruses (i) co-circulate in many parts of the world^19^, (ii) can be transmitted by the same mosquito vectors and (iii) can co-infect both humans^20^ and mosquitoes^21^. Single-construct multi-pathogen vaccines such as the SCV-ZIKA/CHIK vaccine^14^ (and the recently described vesicular stomatitis virus ZIKV/CHIKV vaccine^22^) provide simplified manufacturing and formulation^23^, with multi-pathogen vaccines generally providing reduced ‘shot burden’, increased compliance and reduced costs^1,24^.

Herein we describe the evaluation of a SCV vaccine in NHPs (Indian rhesus macaques, *Macaca mulatta*) and show that SCV-ZIKA/CHIK induces antibody responses to CHIKV, ZIKA and VACV, and provides protection against ZIKV challenge.

## Results

### SCV vaccination

**Fig. 1:**
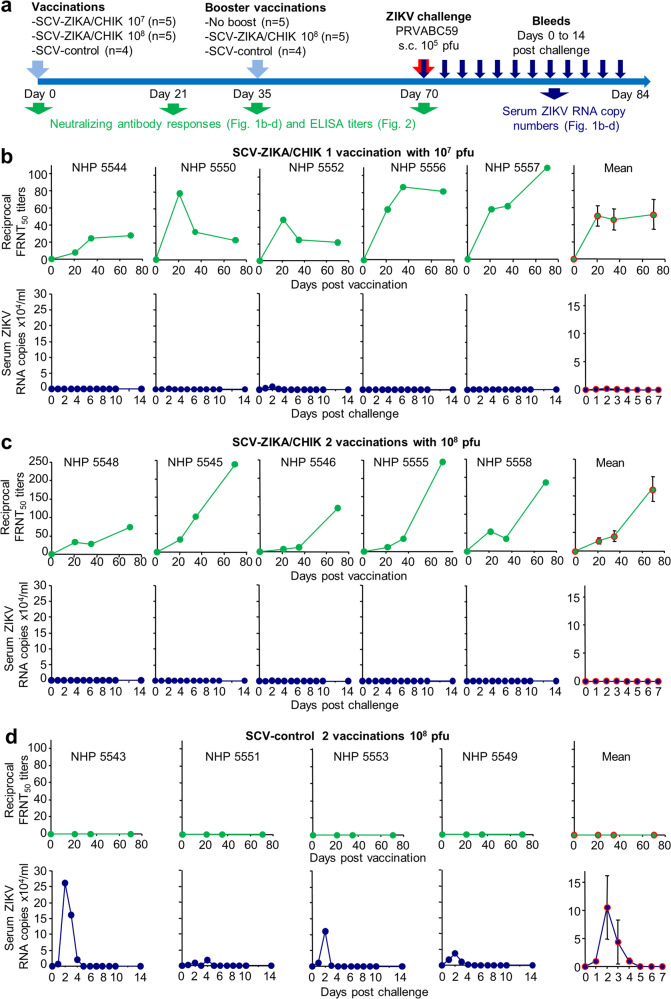
Post-vaccination anti-ZIKV-neutralising antibody responses and ZIKV challenge. **a** Timeline of vaccination, bleeding and challenge. **b** FRNT_50_ titres (green) and serum ZIKV RNA copies/ml (blue) for each NHP at the indicated times after a single vaccination with 10^7^ pfu of SCV-ZIKA/CHIK, with a mean ± SEM graph shown at the far right. ZIKV RNA copies/ml graphs plotted on a log scale and indicating the LLoRQ are provided in Supplementary Fig. 2. **c** As for **b**, after two vaccinations with 10^8^ pfu of SCV-ZIKA/CHIK. ZIKV RNA copies/ml graphs plotted on a log scale and indicating the LLoRQ are provided in Supplementary Fig. 2. **d** As for **b**, after two vaccinations with 10^8^ pfu of SCV-control.

NHPs were vaccinated once with 10^7^ pfu (*n* = 5) or twice with 10^8^ pfu of SCV-ZIKA/CHIK (*n* = 5), or twice with 10^8^ pfu of the SCV-control vaccine (*n* = 4) (Fig. 1a). Animals were vaccinated via an intramuscular injection into the right quadriceps (single site) in a volume of 0.5 ml. No fever or loss of body weight was observed post-vaccination (Supplementary Table 1). No adverse events were noted, except for NHP 5548, who developed a rash by day 35 in the inguinal area, extending from the lower abdomen to the upper thighs. The rash failed to resolve throughout the study and was bilateral, moderately red, flat and similar to those sometimes seen in naive NHPs. When NHPs sit on perches with their legs drawn into their bodies for long periods, moisture and reduced airflow to these areas are likely contributors. This event was thus unlikely to have been related to vaccination, although a rash is an uncommon (≥1/1000 to <1/100) adverse reaction after vaccination of humans with IMVANEX (MVA)^25^.

### ZIKV antibody responses

**Fig. 2:**
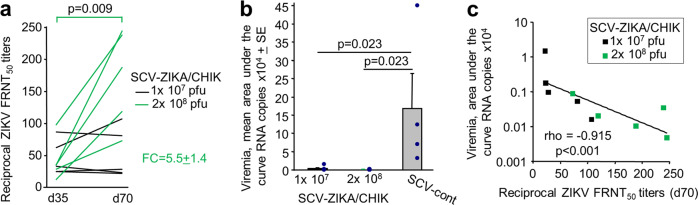
Statistics for ZIKV responses and challenge. **a** Boosting with 10^8^ pfu of SCV-ZIKA/CHIK on day 35 (after the initial vaccination with 10^8^ pfu of SCV-ZIKA/CHIK) resulted in a significant increase in neutralising antibody responses (statistics by paired *t*-test). Each line represents a single NHP. The mean fold change (FC) in titres was 5.5 ± SEM 1.4. For NHPs receiving a single vaccination with 10^7^ pfu of SCV-ZIKA/CHIK on day 0, there was no significant change in titres between day 35 and day 70. **b** SCV-ZIKA/CHIK vaccination provides significant protection against viremia using area under the curve calculations (statistics by Kolmogorov–Smirnov tests). **c** Anti-ZIKV-neutralising antibody responses inversely correlate with ZIKV load. Statistics by Spearman’s rank correlation test.

All NHPs vaccinated with SCV-ZIKA/CHIK generated anti-ZIKV-neutralising antibody responses against ZIKV PRVABC59 (Fig. 1b, c), whereas no significant responses were generated following vaccination with SCV-control (Fig. 1d). A second immunisation with SCV-ZIKA/CHIK at 10^8^ pfu resulted in a significant (*p* = 0.009) increase in the anti-ZIKV neutralising antibody responses, with a fold change (FC) in 50% Focus Reduction Neutralisation Test (FRNT_50_) titres between days 35 and 70 of 5.5 ± SEM 1.4 (Fig. 2a). Titres for NHPs vaccinated with SCV-ZIKA/CHIK at 10^7^ pfu (on day 0) did not change significantly between days 35 and 70 (Fig. 2a). The ZIKV E protein sequence in the SCV-ZIKA/CHIK vaccine (derived from SPH2015, GenBank KU321639) differs by one conservative substitution from the E protein of PRVABC59.

A positive control vaccine comprising formalin-inactivated PRVABC59 given twice intramuscularly (i.m.) to two NHPs generated high levels of neutralising antibody responses to ZIKV PRVABC59 (Supplementary Fig. 1) as described previously^26^.

### ZIKV challenge

All NHPs were challenged with ZIKV PRVABC59 and the ZIKV RNA copy numbers in serum (indicative of viraemias) were determined by quantitative reverse-transcriptase PCR (qRT-PCR) (Fig. 1a). Serum ZIKV RNA copy numbers in both SCV-ZIKA/CHIK-vaccinated groups (Fig. 1b, c) were lower than in the SCV-control vaccine group (Fig. 1d), with both reaching significance using area under the curve (AUC) calculations (Fig. 2b). Two NHPs in the SCV-ZIKA/CHIK 10^7^ pfu vaccine group showed serum ZIKV RNA copy numbers above the lower limit of reliable quantification (LLoRQ) determined to be 860 ZIKV RNA copies/ml, indicating that these animals had low level viremias. These animals were NHP 5552 that exceeded the LLoRQ for 3 days and NHP 5550 that exceeded the LLoRQ for 1 day (Supplementary Fig. 2). These two NHPs also had the lowest neutralising titres of the SCV-ZIKA/CHIK-vaccinated animals on the day of challenge (Fig. 1b, Day 70). None of the NHPs vaccinated with 2 × 10^8^ pfu of SCV-ZIKA/CHIK showed serum ZIKV RNA copy numbers above the LLoRQ, whereas all NHPs receiving the SCV-control vaccine showed serum ZIKV RNA copy numbers above the LLoRQ (Supplementary Fig. 2). As expected^26^, the two NHPs vaccinated with formalin-inactivated ZIKV PRVABC59 vaccine showed no serum ZIKV RNA copy numbers above the LLoRQ (Supplementary Figs. 1 and 2).

Notwithstanding the uncertainties of qRT-PCR determinations below the LLoRQ, for all SCV-ZIKA/CHIK-vaccinated NHPs (*n* = 10), the AUC for ZIKV RNA copies/ml (Supplementary Fig. 2) inversely correlated (*p* < 0.001) with neutralisation titres (Fig. 2c). This analysis supports the view that neutralising antibodies play a protective role against ZIKV infection^27^, although it does not preclude a protective role for T cells^28,29^.

### CHIKV antibody responses

Three CHIKV genotypes are recognised; the West African (WA); the East, Central and South African (ECSA), and the Asian. An Indian Ocean sub-lineage (IOL) arose from the ECSA lineage during the Indian Ocean outbreak. Asian genotype viruses (introduced into the Caribbean in 2013) were responsible for most of the disease in the Americas, with the American viruses forming a sub-lineage within the Asian genotype^30^. (ECSA genotype viruses were recently identified in the Americas^31^). The NHP sera were thus analysed for their ability to neutralise four representative CHIKV isolates (i) LR2006-OPY1, a 2006 IOL isolate from Reunion Island (GenBank: DQ443544; resequenced KT449801.1), (ii) 37997, a 1983 WA isolate from Senegal (GenBank: AY726732.1), (iii) R99659, a contemporary 2014 Asian genotype virus from the British Virgin Islands in the Caribbean (GenBank: KX713902.1/KJ451624.1) and (iv) AF15561, a 1962 Asian genotype isolate from Thailand (GenBank: EF452493.1). The CHIKV antigen in the SCV-ZIKA/CHIK vaccine comprises the structural polyprotein cassette of the 2006 IOL isolate, 06–021, from Reunion Island (GenBank; AM258992)^13,14^. A dendogram of the E2 protein sequences of these isolates is shown in Supplementary Fig 3. E2 is a major target of neutralising antibodies^32–34^

**Fig. 3:**
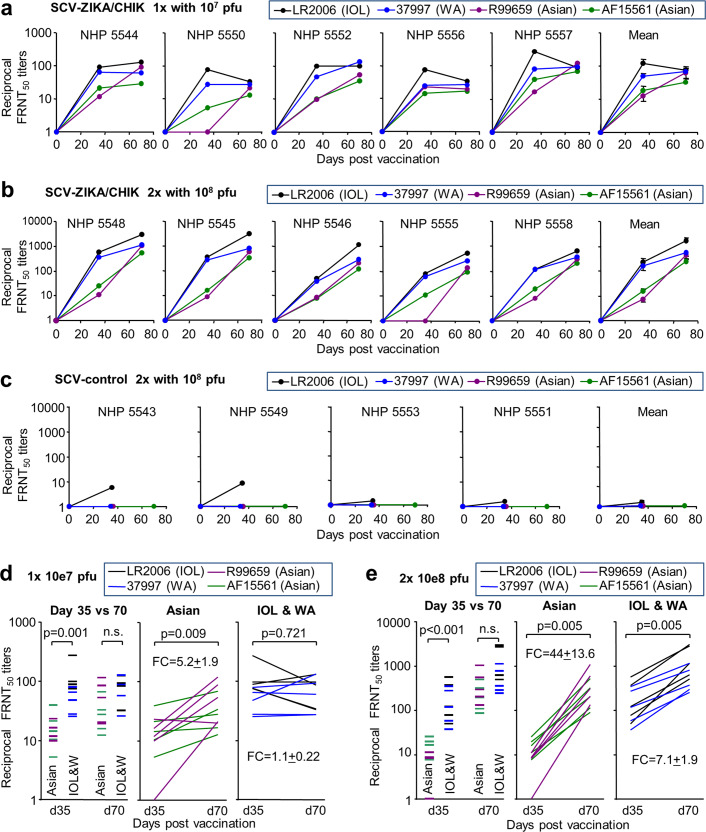
Post-vaccination anti-CHIKV -neutralising antibody responses. **a** FRNT_50_ titres against each of the four CHIKV isolates for each NHP at the indicated times after a single vaccination with 10^7^ pfu of SCV-ZIKA/CHIK, with mean ± SEM titres shown far right. **b** As for **a**, after two vaccinations with 10^8^ pfu of SCV-ZIKA/CHIK. **c** As for **a**, after two vaccinations with 10^8^ pfu of SCV-control. For **a**–**c**, full titration curves are shown in Supplementary Fig. 4. **d** For NHPs vaccinated with 10^7^ pfu of SCV-ZIKA/CHIK the day 35 and 70 neutralising antibody titres are plotted. All isolates, 20 lines for 4 CHIKV isolates and 5 NHPs. Asian, 10 lines for two Asian isolates and 5 NHPs. IOL and WA, 10 lines for LR2006 and 37997 isolates, and 5 NHPs. The mean fold change (FC) ± SEM is provided at the bottom right. Statistics by related samples Wilcoxon signed-rank tests. **e** As for d but for two vaccinations (days 0 day 35) with 10^8^ pfu of SCV-ZIKA/CHIK.

SCV-ZIKA/CHIK vaccination resulted in the generation of neutralising antibodies to all four CHIKV isolates (Fig. 3a–b), whereas responses following SCV-control vaccination were minimal (Fig. 3c). The individual titration curves are shown in Supplementary Fig 4.

On day 35 after one vaccination with 10^7^ pfu of SCV-ZIKA/CHIK, the responses to the two Asian isolates (R99659 and AF15561) were 5.6-fold lower than those against the IOL and WA isolates (Fig. 3d, Day 35 vs. 70). Between days 35 and 70, the responses to the Asian isolates increased significantly by a mean of 5.2-fold (*p* = 0.009) (Fig. 3d, Asian), whereas the responses to the IOL and WA isolates did not change significantly (Fig. 3d, IOL and WA). Similarly, for the 2 × 10^8^ pfu group, neutralising antibody responses to the Asian isolates were a mean of 30-fold lower than the IOL and WA isolates on day 35 (*p* < 0.001), but on day 70 they were not significantly different (Fig. 3e, Day 35 vs. 70). Between days 35 and 70, the responses to the Asian isolates increased significantly by a mean of 44 fold (*p* = 0.005) (Fig. 3e, Asian), whereas the responses to the IOL and WA isolates increased by 7.1-fold (Fig. 3e, IOL and WA). Thus, by day 70 (irrespective of the vaccine dose and schedule), responses to all the CHIKV isolates were not significantly different. However, neutralising antibody responses to the Asian isolates rose less quickly between days 0 and 35, but rose more rapidly between day 35 and 70, than neutralising antibody responses to the IOL and WA isolates.

### VACV antibody responses

**Fig. 4:**
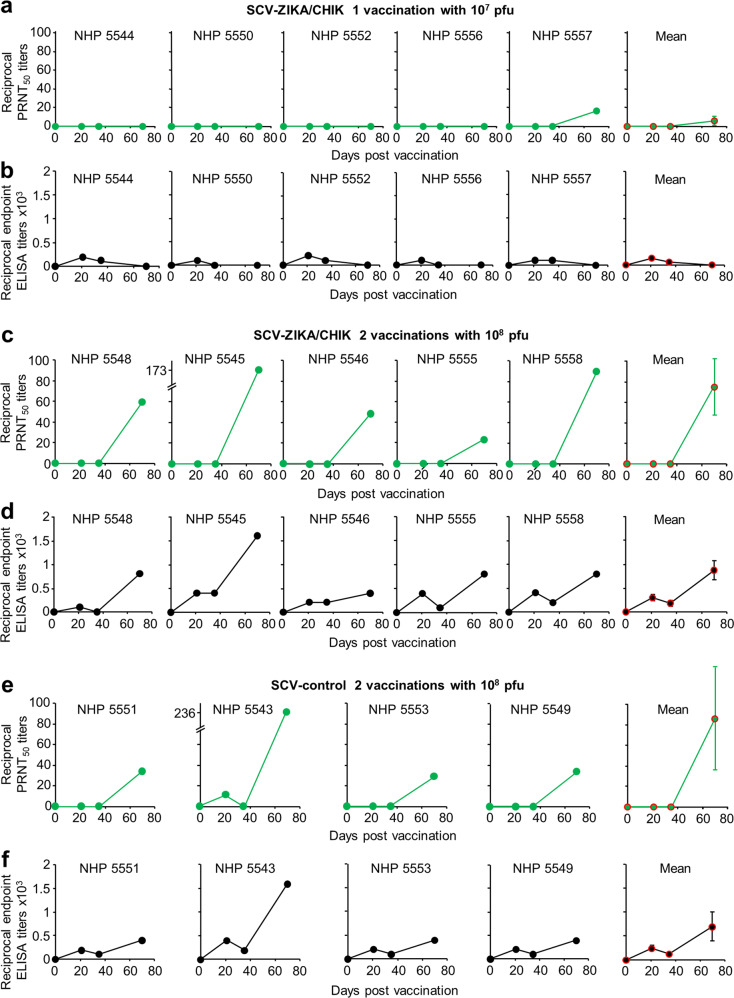
Post-vaccination anti-VACV responses. **a** Reciprocal PRNT_50_ (neutralising antibody) titres against VACV (Western Reserve) for each NHP at the indicated times after a single vaccination with 10^7^ pfu of SCV-ZIKA/CHIK, with mean ± SEM titres shown far right. Limit of detection one in ten serum dilution. **b** Reciprocal endpoint ELISA anti-VACV (Western Reserve) antibody titres for each NHP at the indicated times after a single vaccination with 10^7^ pfu of SCV-ZIKA/CHIK, with mean ± SEM titres shown far right. Limit of detection 1 in 100 serum dilution. **c** Reciprocal PRNT_50_ titres as for a after two vaccinations with 10^8^ pfu of SCV-ZIKA/CHIK. **d** Reciprocal endpoint ELISA titres as for **b** after two vaccinations with 10^8^ pfu of SCV-ZIKA/CHIK. **e** Reciprocal PRNT_50_ titres as for **a** after two vaccinations with 10^8^ pfu of SCV-control. **f** Reciprocal endpoint ELISA titres as for **b** after two vaccinations with 10^8^ fu of SCV-control (no antibody responses to VACV were detected in NHPs 5547 and 5554 that received the inactivated ZIKV vaccine).

We have previously shown in mice the induction of anti-VACV antibody responses by SCV vaccination and protection against ectromelia virus (mouse pox) challenge^13^, suggesting the potential for SCV to also be used as a smallpox vaccine. The VACV neutralising (Fig. 4a, c, e) and IgG enzyme-linked immunosorbent assay (ELISA) antibody (Fig. 4b, d, f) titres induced by SCV vaccination were thus determined. A single vaccination with 10^7^ pfu only produced detectable neutralising antibody responses in 1 NHP (NHP557), with low IgG ELISA response seen on days 20 and/or 40, but below detection by day 70 post vaccination (Fig. 4a–b). After 2 i.m. vaccinations with 10^8^ pfu of SCV vaccines (given week 0 and 5), the VACV neutralisation titre reached a mean reciprocal 50% Plaque Reduction Neutralisation test (PRNT_50_) titre of 79 ± SEM 24 (*n* = 9) by day 70, taking both the 2 × 10^8^ SCV-ZIKA/CHIK (Fig. 4c) and SCV-control groups (Fig. 4e) together. The VACV ELISA IgG titres largely paralleled the neutralising antibody responses, with both the 2 × 10^8^ SCV-ZIKA/CHIK (Fig. 4d) and the SCV-control groups (Fig. 4f) reaching similar mean titres by day 70 with an overall (both groups) mean reciprocal endpoint ELISA titre of 800 ± SEM 163 (*n* = 9).

## Discussion

We present herein a NHP immunogenicity study for an SCV vaccine. SCV-ZIKA/CHIK vaccination induced anti-CHIKV, anti-ZIKV and anti-VACV antibody responses and mediated protection against ZIKV challenge.

We show herein that SCV-ZIKA/CHIK vaccination was able to reduce post-challenge serum ZIKV RNA copy numbers to levels below those that can reliably be detected, except in two NHPs that received one dose of 10^7^ pfu SCV-ZIKA/CHIK (NHPs 5552 and 5550). Sterilising immunity (nominally defined as no viral RNA detected) was not achieved for any NHP, even those vaccinated with the positive control vaccine^26^ (Supplementary Fig. 2). Whether sterilising immunity is required to prevent transplacental fetal brain infection in pregnant women remains unclear. Whether low levels of placental or fetal infection could give rise to neonatal disabilities, or disabilities that might manifest later in life, remains similarly unclear^35^. The highly sensitive nature of qRT-PCR would make such sterilising immunity difficult to achieve, with very high levels of neutralising antibodies likely required^36^. Attaining and sustaining such high levels in women of child-bearing age would represent a considerable challenge, especially given the additional safety concerns in this population. A consensus view will have to await vaccine studies in pregnant NHPs, with the a hope being that a vaccine that is able to reduce substantially ZIKV replication will largely prevent or significantly ameliorate the rate and severity of CZS. Such a hope is supported by the success of vaccination campaigns against rubella^37^ (family *Matonaviridae*, genus *Rubivirus*) and bovine viral diarrhoea virus (family *Flaviviridae*, genus *Pestivirus*)^38^.

A concern for ZIKV vaccines is that they may induce antibody-dependent enhancement (ADE) of dengue virus (DENV)^39^, although no good evidence for such activity, or for ADE of ZIKV via prior DENV infection^40^, has emerged from studies of natural infections in humans^41^. Nevertheless, a poorly designed vaccine may (even if natural infections do not) provide opportunities for ADE. Conceivably, given recent insights, regulators may require mutation of the fusion loop region^39^ and/or the prM cleavage site^42^ to obviate the risk of ADE. Such modifications could be readily incorporated into recombinant vaccines such as SCV-ZIKA/CHIK, but might be less straightforward for live attenuated or inactivated whole-virus ZIKV vaccines (where viral replication is required in the vaccine recipient or during manufacture). The assay systems illustrating the absence of ADE that would satisfy regulators remain unclear, with in vitro assays perhaps now viewed as less reliable^41^, given inter alia the widespread ability to show ADE in vitro for a range of different viral genera^43^. We have established an IFNAR1^−/−^ mouse model that shows ADE of DENV disease and were able to show that SCV-ZIKA/CHIK vaccination did not enhance DENV disease in this model (Supplementary Fig. 5). Although encouraging, the requirements for ZIKV vaccines in this space will have to await vaccine studies in pregnant NHPs and the development of consensus views by regulators.

Although all CHIKV strains are thought to belong to the same serogroup^44^, differences in cross neutralisation between isolates from different genotypes have been reported^45^. The data presented herein suggests increased time post-vaccination increases the cross-neutralising capacity against multiple CHIKV genotypes. Although many neutralising responses are directed at tertiary or quaternary structures^46,47^, a linear epitope (E2EP3) at the N terminus of the E2 protein was identified as the target of many early neutralising responses in CHIKV-infected patients and NHPs^32^. The sequence of this epitope is identical in the CHIKV strain encoded by the SCV vaccine (IOL CHIKV isolate 06–021), the IOL isolate (LR2006-OPY1) and the WA isolate (37997), whereas it has 1 or 2 non-conservative substitutions in the Asian isolates (R99659 and AF15561) (Supplementary Fig. 6). Antibody responses present later in CHIKV infections were found to be less focused on this epitope^32^, suggesting a level of epitope spreading over time. Early E2EP3-focused responses may explain why on day 35 SCV-ZIKA/CHIK induced higher neutralising responses to the IOL and WA isolates (conserved E2EP3 sequences). Later (day 70), epitope spreading may ensure similar levels of cross-neutralising responses to all the genotypes (Fig. 3a, b Mean). Similar broadening of responses has been shown to be promoted by adjuvanting of influenza vaccines^48,49^. The development of broadly neutralising antibodies over several years has also been reported in HIV patients^50^. The data presented herein is a clear illustration of time-dependence for the post-vaccination development of broadly neutralising responses against different CHIKV genotypes.

Precisely, what level of neutralising antibody response is needed to protect against CHIKV is not entirely clear, although a conservative correlate of protection was defined as a reciprocal 50% neutralising serum antibody titre of a ≥10 in a phase 3 trial of a vaccine against the related Ross River virus^51^. In a rapidly lethal interferon signalling-deficient A129 mouse model, a minimum reciprocal 50% neutralising antibody titre of 35 conferred complete protection against CHIKV challenge^52^. After a single vaccination of NHPs herein with 10^7^ pfu of SCV-ZIKA/CHIK, the mean day 70 reciprocal FRNT_50_ anti-CHIKV titre was 59.4 ± SEM 8.9 (range 28–93) (*n* = 20; 5 NHPs and 4 CHIKV strains). After two vaccinations with 10^8^ pfu, this value was 736 ± SEM 187 (range 90–3090). SCV-ZIKA/CHIK vaccination thus induced neutralising antibody responses to CHIKV that might be expected to provide protection against CHIKV challenge.

The mean SCV-induced anti-VACV reciprocal PRNT_50_ neutralisation titre of 79 ± SEM 24 after 2 vaccinations i.m. with 10^8^ pfu of SCV vaccines compares favourably with phase III human trial data (i) where MVA vaccination resulted in a mean reciprocal PRNT_50_ neutralisation titre of 94 (95% confidence interval (95% CI) 78–113) (*n* = 189–220) on day 84 after 2 subcutaneous (s.c.) vaccinations (given weeks 0 and 4) with 10^8^ TCID_50_ of MVA and (ii) where vaccination with the live VACV vaccine, ACAM2000, produced a mean reciprocal PRNT_50_ neutralisation titre of 65.6 (95% CI 54–79) (*n* = 189–220) on day 56 after one vaccination by killing^8^. Although assay systems might differ, both herein and in the study by Pittman et al.^8^, the Western Reserve strain of VACV was used for the antibody assays. The immune correlates of protection against smallpox are not well characterised, with both CD8 T cells and neutralising antibodies thought to play a role^53^. Smallpox (and VACV) exists in two forms: intracellular mature virions (MVs) with one envelope and extracellular enveloped viruses that have an additional envelope. Antibodies directed at both virion forms are believed to be required for optimal protection^54,55^; however, the VACV used for neutralisation assays usually comprises primarily MVs. The results in Fig. 4 thus suggest that SCV may find utility as a smallpox vaccine; however, NHP challenge studies using monkeypox virus are needed to support this contention.

A number of limitations of this study are recognised. The limited available budget precluded a more extensive dose ranging and scheduling series for the SCV-ZIKA/CHIK vaccine, with only one arguably low end (one vaccination with 10^7^) and one high end (two vaccination with 10^8^ pfu) dosing/scheduling tested. In addition, although poxvirus vaccine systems generally show considerable utility for induction of T-cell responses^1^, these were not accessed in this study, with both CD4 and CD8 T cells likely mediating protection against ZIKV^28,29^. No CHIKV challenge was undertaken due to the high costs associated with NHP studies under BSL3 conditions; furthermore, NHP models are currently also unable to provide arthralgia or overt arthritis readouts^56,57^. Nevertheless, CHIKV neutralising antibody titres were reached that might be expected to provide protection from vireamia and athropathy. We were also unable to gain access to any Brazilian ECSA isolates^58^ for use in this industry-supported project; however, such isolates show lower levels of divergence from the vaccine strain than the Asian isolates (to which neutralising response were generated).

In summary, the results of this NHP study of the SCV technology support the view that SCV-ZIKA/CHIK vaccination can induce immunity in NHPs that is sufficient for protection against ZIKV and CHIKV. Perhaps most encouraging was the ability of a single vaccination and a homologous prime-boost to induce significant anti-ZIKV and anti-CHIKV-neutralising antibody responses in NHPs, potentially obviating the need for heterologous prime-boost strategies for recombinant SCV vaccines.

## Methods

### Care, use of animals and AEC approval

The study design was reviewed by the Institutional Animal Care and Use Committee at Southern Research (Internal Approval #18–03–008 F; AAALAC Accreditation #000643; OLAW Assurance D16–00025-Legacy #A3046–1). All animals were cared for and procedures performed in accordance with the institutional guidelines for the care and use of experimental animals, abiding ethical regulations for animal testing and research. Animals were socially housed during the quarantine and pre-study phases, then single-housed following ZIKV challenge. Animals were housed in stainless steel cages that meet requirements set forth in the Animal Welfare Act (Public Law 99–198, USA) and the Guide for the Care and Use of Laboratory Animals (8th Edition, Institute of Animal Resources, Commission on Life Sciences, National Research Council, National Academy Press, Washington DC, 2011). Animals were housed in environmentally monitored and ventilated rooms. Fluorescent lighting provided illumination approximately 12 h per day. Indian rhesus macaques *M. mulatta* were supplied by PrimGen (Hines, IL, USA), were aged between 1 and 6 years, and weighed between 2.0 and 8.0 kg. A total of 16 animals (8 females and 8 males) were used. All animals were placed into quarantine in the ABSL-2 facility for a minimum of 30 days prior to ZIKV challenge. All animals were screened and confirmed to be free of antibodies to simian immunodeficiency virus, simian retroviruses, simian T-cell leukaemia virus type 1 and Herpes B virus. Animals were also tested and confirmed to be negative for tuberculosis, klebsiella, *Trypanosoma cruzi*, West Nile virus, DENV and ZIKV.

Macaques were fed twice per day with Purina LabDiet 5048 Certified NHP Diet during the quarantine and study periods. Analyses of the feed, provided by the manufacturer were reviewed by the veterinarian to ensure that no known contaminants were present that could interfere with, or affect, the outcome of the study. In addition, as part of the normal diet, animals were given a variety of fruit and vegetables. Animals were monitored for decreased appetite and/or significant weight loss. All animals were given a unique identification number via a tattoo and were observed twice daily throughout the quarantine and study periods for signs of morbidity and mortality. During the challenge period (day 70–84), animals were observed twice daily for responsiveness and clinical signs including rash, erythema, conjunctivitis, ocular discharge and swelling. Rectal temperatures and body weights of each animal were measured prior to blood collection.

### Vaccination and challenge

Prior to study initiation, Indian Rhesus macaques were randomised into respective groups according to gender and weight using Provantis Software (Instem, USA). SCV-ZIKA/CHIK 10^7^ pfu had two males and three females; SCV-ZIKA/CHIK 2 × 10^8^ pfu had three males and two females; SCV-control had two males and two females; inactivated PRVBC59 had one male and one female. Prior to vaccination, bleeding and challenge, animals were anaesthetized using ketamine hydrochloride administered i.m. at 5–30 mg/kg in a volume of 1 ml or less per site. Vaccines were administered i.m. into the right quadriceps (0.5 ml single site), with animals receiving SCV-ZIKA/CHIK or SCV-control (or a positive control formalin-inactivated reference PRVABC59 vaccine^26^).

Animals were challenged s.c. (anterior surface of the left forearm) with 0.5 ml of wild-type ZIKV strain PRVABC59 (10^5^ pfu per animal). Prior to all injections, the injection site was clipped, wiped with alcohol and marked with an indelible marker. Bloods were collected into Serum Separator tubes (2–8 ml) and serum was aliquoted and stored at −80 °C.

### SCV vaccines and their production

The SCV-ZIKA/CHIK and SCV-control vaccines have been described previously^14^. The SCV-ZIKA/CHIK vaccine encodes from two separate loci: (i) the CHIKV structural polyprotein cassette (C-E3-E2–6K-E1) from a 2006 isolate from Reunion Island (strain 06–021) (Genebank: AM258992)^13^, which is 1249 amino acids (3747 bp) in length (GenBank: CAJ90476.1) and (ii) ZIKV prME from a 2015 Brazilian isolate (ZikaSPH2015) (Genbank: KU321639)^14^, which is 692 amino acids (2067 bp) in length (GenBank: ALU33341.1).

The SCV-ZIKA/CHIK and SCV-control vaccines were produced in a non-GMP BSL2 SCS line (comprising CHO-S cells transfected with D13L and CP77^13^) using serum and protein-free cell culture conditions. The vaccines were purified by centrifugation through a sucrose cushion. Briefly, infected cells were harvested by centrifugation. Cell-associated virus was released using multiple freeze–thaw cycles in 10 mM Tris HCl pH 8.0 and 150 mM NaCl. Viral extracts were centrifuged to remove the majority of cell debris. The clarified extract was further purified by centrifugation through a 36% sucrose cushion. The viral pellets were resuspended in 10 mM Tris HCl pH 8, 150 mM NaCl buffer and stored frozen at −80 °C.

PCR analyses confirmed the presence of the CHIK and the ZIKA expression cassettes and absence of wild-type SCV^14^. Sterility testing of the purified SCV vaccines was adapted from FDA-CBER 21 CFR 610.12 and comprised inoculation of Thioglycollate Medium and Soybean-Casein Digest Medium and turbidity testing. Mycoplasma testing was performed using Mycoplasma PCR ELISA kit (Roche Applied Science, catalogue number 11 663 925 910).

Single-use tubes were filled with SCV vaccines at 10^8^ pfu per 500 μl with 100 μl over-fill. Vaccines were shipped to Southern Research Fredrick USA on dry ice. Mock transport was carried out by maintaining a separate set of vaccines on dry ice for the same period, with minimal loss of titres.

### Preparation of ZIKV for NHP challenge

ZIKV strain PRVABC59 was isolated in 2015 from human serum collected in Puerto Rico and obtained from the Centers for Disease Control and Prevention (Division of Vector-borne Infectious Diseases, CDC, Fort Collins, CO, USA). Virus was amplified in Vero cells (CCL-81, ATCC, Manassa, VA), quantified using standard plaque assay on Vero cells (PFU/ml), with all stocks tested for mycoplasma, endotoxin (<0.1 EU/ml) and sterility.

### ZIKV Focus Reduction Neutralisation Test (FRNT_50_)

The FRNT_50_ assay for ZIKV was performed on NHP serum samples. Briefly, fourfold serially diluted serum samples and ZIKV (strain PRVABC59) (≈100 pfu/ml) were incubated for 1 h at 37 °C and added to Vero cells, with overlay medium added after 1 h. After overnight culture, the 96-well plates were stained with an anti-flavivirus monoclonal antibody (MAB10216, Millipore, Burlington, MA USA) and goat anti-mouse IgG (H + L) horseradish peroxidase (HRP)-conjugated secondary antibody (5220–0341, SeraCare Life Sciences, Milford, MA, USA). TrueBlue Peroxidase Substrate (5510–0030, SeraCare Life Sciences, Milford, MA, USA) was added to the plate and spots counted by CTL Biospot Analyzer and Biospot software (Cellular Technology, Ltd, Cleveland, OH, USA). FRNT_50_ titres were calculated by interpolation between the two points that spanned 50% neutralisation.

### Serum viral load determination by qRT-PCR

qRT-PCR was performed using RNA isolated from serum. Briefly, RNA was extracted from serum samples using QIAmp Viral RNA Mini kit (Qiagen, 52906, Germantown, MD, USA) and analysed in triplicate by qRT-PCR using primers directed to the envelope protein (forward primer 5′-TGAGGCATCAATATCAGACATG-3′ and reverse primer 5′-GTTCTTTTGCAGACATATTGAGTG-3′). Five microlitres of purified RNA from each sample was used in a 20 µL qRT-PCR reaction consisting of Fast Virus 4× Master Mix (Applied Biosystems, 4444436, Foster City, CA, USA) containing 500 nM forward and reverse primers with a 200 nM probe. Cycling parameters include the following: an initial reverse transcription step for 5 min at 53 °C, followed by 1 min at 95 °C and 45 cycles of two-step cycling at 95 °C for 5 s and 60 °C for 50 s. Data were expressed as viral genome copies/ml using a standard curve established using quantified in vitro-transcribed viral RNA. The LLoRQ was 860 viral genome copies/ml of serum. The LLoRQ represents the lowest limit of 100% reliable quantification over a large number of qRT-PCR experiments using serum samples spiked with in vitro*-*transcribed ZIKV RNA.

### CHIKV 50% Focus Reduction Neutralisation Test (FRNT_50_)

NHP serum samples were sent frozen on dry ice to University of Colorado School of Medicine and were thawed and heat inactivated. A FRNT_50_ assay was used as described by Hawman et al.^59^. NHP sera were serially diluted in duplicate in Dulbecco’s modified Eagle’s medium (DMEM)/F:12 medium (Gibco) plus 2% fetal bovine serum (FBS) in 96-well plates (2-fold serial dilutions starting at 1 : 8 dilution). Approximately 100 focus-forming units of the indicated CHIKV isolate were added to each well and the serum plus virus mixture was incubated for 1 h at 37 °C. The mixtures were then added for 2 h at 37 °C to parallel 96-well plates seeded with Vero cells. The mixtures were then removed and cells were overlaid with 0.5% methylcellulose in MEM/5 % FBS and incubated 18 h at 37 °C. Cells were then fixed with 1% paraformaldehyde and probed with 500 ng/ml of the anti-CHIKV monoclonal antibody, CHK-11^60^ in wash buffer (1× phosphate-buffered saline (PBS)/0.1% saponin/0.1% bovine serum albumin) for 2 h at room temperature. After washing, cells were incubated with horseradish peroxidase-conjugated goat anti-mouse IgG (Southern Biotech, Birmingham, AL, USA), diluted 1 : 2000 for 1.5–2 h at room temperature. After washing, CHIKV-positive foci were visualised with TrueBlue peroxidase substrate (SeraCare, Milford MA, USA) and counted using a CTL Biospot Analyzer and Biospot software. The FRNT_50_ titre was calculated relative to a virus only (no NHP anti-serum) control set at 100%, using GraphPad Prism 7 (La Jolla, CA, USA) default nonlinear curve fit constrained between 0 and 100%. CHIKV work was conducted in the BSL3 facility at University of Colorado School of Medicine and was approved by institutional biosafety committee (No 09–003).

LR2006-OPY1 (GenBank: DQ443544; resequenced KT449801.1) was recovered from a DNA clone provided by Dr S. Higgs (University of Texas Medical Branch, Galveston, Texas, USA), 37997 (GenBank: AY726732.1) was provided as a virus by Dr Ann Powers (Division of Vector-Borne Diseases, CDC, Atlanta, GA, USA), R99659 (GenBank: KX713902.1/KJ451624.1) was recovered from a DNA clone provided by Dr M.T. Heise (University of North Carolina at Chapel Hill, Chapel Hill, NC, USA) and AF15561 (GenBank: EF452493.1) was recovered from a DNA clone provided by Dr T.S. Dermody (University of Pittsburgh School of Medicine, Pittsburgh, USA).

### VACV Plaque Reduction Neutralisation test (PRNT_50_) assays

The ability of vaccine-induced antibodies to neutralise VACV was evaluated in a validated VACV-specific PRNT_50_ assay in accordance with Southern Research standard operating procedures. Briefly, serum samples were serially diluted in DMEM containing Glutamax and 2% FBS, and added to an equal volume of a fixed dilution of VACV (Western Reserve strain). The serum-virus mixture was then incubated overnight at 2–8 °C. Subsequently, 100 µl of each serum-virus mixture was added, in triplicate, to a fresh 24-well plate containing confluent Vero cells and incubated at 37 ± 1 °C. VACV pre-incubated with normal monkey or vaccinia immune globulin polyclonal antibody serving as negative and positive controls, respectively. Neutralisation endpoint titres were calculated based on the reciprocal dilution of the test serum that produced 50% plaque reduction compared with the virus control.

### VACV ELISA assays

To measure VACV-specific antibody responses, serum samples were analysed using a validated VACV-specific ELISA in accordance with Southern Research Frederick Standard Operating Procedure. Briefly, 96-well ELISA plates were coated with 100 µl/well of 0.5 µg/ml purified VACV (Western Reserve strain) antigen in PBS and incubated overnight at 2–8 °C. Plates were washed using an automated plate washer (BioTek ELx405, Winooski, VT) and then blocked with 5% non-fat milk in 0.05% PBS Tween 20 (PBST) at 37 ± 1 °C. Serum samples were serially diluted in triplicate in 5% non-fat milk in 0.05% PBST and added at 100 µl/well. Specific antibodies were detected using a goat anti-monkey HRP-conjugated IgG (Sigma-Aldrich) as secondary antibody. Plates were developed with 100 µl/well of ABTS substrate for 15–20 min, stopped with 100 µl of 1% SDS in distilled water and read at 405 nm using a SpectraMax plate reader (Molecular Devices. Sunnyvale, CA). Positive (monkey polyclonal antibody) and negative (normal Serum) control samples were included on each plate.

### Statistics

Statistics were performed using IBM SPSS Statistics (version19). The two-sided *t*-test was used if the difference in the variances was <4, skewness was > −2 and kurtosis was <2, where the data was nonparametric and difference in variances were >4, the Kolmogorov–Smirnov test was used. The related samples Wilcoxon signed-rank test was used for paired, nonparametric data. Correlations were analysed using the nonparametric Spearman’s rank correlation test.

### Reporting summary

Further information on research design is available in the Nature Research Reporting Summary linked to this article.

## Supplementary information

Supplementary Information

Reporting Summary

## Data Availability

All datasets used and/or analysed in the current study are available from the corresponding author upon reasonable request.
