# Supplementary material for: The vaccinia virus based Sementis Copenhagen Vector vaccine against Zika and chikungunya is immunogenic in non-human primates
Source: NPJ Vaccines. 2020 Jun 2;5:44. doi: 10.1038/s41541-020-0191-8 (PMC7265471; doi:10.1038/s41541-020-0191-8)
Supplement: Supplementary file 1 — Supplementary Information [file 41541_2020_191_MOESM1_ESM.pdf]

Supplementary Table 1. NHP rectal temperatures and body weights post vaccination and challenge. Vaccinations days 0 and 35, challenge day 70.

Body temperature (F) (deg. F)

Group	Animal	Day numbers relative to Start Date: 0	21	35	70	71	72	73	74	75	76	77	78	79	80	84
1x 10^7 pfu SCV-Z/C	5544	102.3	101.9	101.6	102.4	100.8	101.1	101.2	100.6	100.3	100.7	102.3	100.9	101.2	100.7	101.6
	5550	102.7	102.3	102.8	102.4	102.2	101.1	100.1	101.0	102.4	101.8	101.3	102.0	102.3	101.5	101.5
	5552	102.0	102.8	101.1	102.3	102.2	101.0	101.7	101.4	100.7	102.0	100.5	101.1	100.5	101.7	101.6
	5556	103.8	102.5	102.3	102.7	100.8	100.4	101.4	103.4	101.9	100.4	101.7	101.5	101.2	102.4	102.2
	5557	101.9	101.8	102.2	101.8	100.8	100.9	101.5	101.9	100.7	102.0	101.3	101.0	101.2	101.2	100.9
	Mean	102.54	102.26	102.00	102.32	101.36	100.90	101.18	101.66	101.20	101.38	101.42	101.30	101.28	101.50	101.56
	S.D.	0.77	0.42	0.66	0.33	0.77	0.29	0.63	1.09	0.90	0.77	0.66	0.45	0.65	0.63	0.46
	N	5	5	5	5	5	5	5	5	5	5	5	5	5	5	5

Group	Animal	Day numbers relative to Start Date: 0	21	35	70	71	72	73	74	75	76	77	78	79	80	84
2x 10^8 pfu SCV-Z/C	5548	101.4	101.6	101.1	101.9	101.8	101.2	100.6	100.7	100.2	101.7	100.8	100.3	100.8	100.3	100.1
	5545	101.5	101.8	101.1	100.4	101.3	102.0	101.9	100.3	99.3	100.5	100.5	101.6	100.6	102.6	101.8
	5546	101.7	101.5	101.1	101.9	101.4	101.7	100.1	100.8	100.6	101.9	101.5	101.3	101.3	100.9	101.7
	5555	102.9	102.1	102.0	102.7	101.7	99.5	100.8	101.2	102.0	99.2	102.1	100.3	101.7	102.2	101.6
	5558	102.0	100.7	101.9	100.8	101.0	100.8	101.2	102.0	101.4	101.0	101.0	100.8	101.9	98.9	99.5
	Mean	101.90	101.54	101.44	101.54	101.44	101.04	100.92	101.00	100.70	100.86	101.18	100.86	101.26	100.98	100.94
	S.D.	0.60	0.52	0.47	0.93	0.32	0.98	0.68	0.64	1.05	1.08	0.63	0.59	0.56	1.49	1.06
	N	5	5	5	5	5	5	5	5	5	5	5	5	5	5	5

Group	Animal	Day numbers relative to Start Date: 0	21	35	70	71	72	73	74	75	76	77	78	79	80	84
2x 10^8 pfu SCV-cont	5543	103.4	102.0	102.3	101.2	101.3	100.3	103.8*	101.5	101.5	101.0	102.0	101.9	100.2	102.1	101.4
	5549	102.3	101.9	102.0	102.0	101.2	101.3	100.2	101.5	102.0	100.9	101.4	101.7	102.5	102.3	100.8
	5553	102.1	102.9	101.0	101.9	100.8	101.5	101.2	100.9	101.4	100.7	101.3	102.7	101.3	101.6	102.4
	5551	101.8	102.6	102.2	103.1*	102.4	99.8	101.3	100.2	101.2	101.7	101.2	100.2	101.9	102.8	103.2
	Mean	102.40	102.35	101.88	102.05	101.43	100.73	101.63	101.03	101.53	101.08	101.48	101.63	101.48	102.20	101.95
	S.D.	0.70	0.48	0.60	0.79	0.68	0.81	1.53	0.62	0.34	0.43	0.36	1.04	0.98	0.50	1.06
	N	4	4	4	4	4	4	4	4	4	4	4	4	4	4	4

Bodyweight (kg)

Group	Animal	Day numbers relative to Start Date: 0	21	35	70	71	72	73	74	75	76	77	78	79	80	84
1x 10^7 pfu SCV-Z/C	5544	5.5	5.6	6.0	6.1	6.1	6.0	6.0	6.2	6.2	6.1	6.2	6.1	6.1	6.2	6.2
	5550	2.3	2.4	2.5	2.6	2.6	2.6	2.5	2.6	2.5	2.5	2.5	2.5	2.5	2.5	2.6
	5552	4.1	4.0	4.1	3.9	3.9	3.8	3.8	3.8	3.9	3.9	3.9	3.9	3.9	4.0	4.0
	5556	2.2	2.3	2.4	2.4	2.4	2.3	2.4	2.3	2.3	2.4	2.3	2.4	2.4	2.6	2.5
	5557	5.7	6.0	6.2	6.0	5.9	5.9	5.9	6.0	6.0	6.0	5.9	5.9	6.1	6.3	6.1
	Mean	3.96	4.06	4.24	4.20	4.18	4.12	4.12	4.18	4.18	4.18	4.16	4.16	4.20	4.32	4.28
	S.D.	1.68	1.73	1.83	1.78	1.76	1.76	1.76	1.84	1.86	1.81	1.84	1.78	1.83	1.86	1.81
	N	5	5	5	5	5	5	5	5	5	5	5	5	5	5	5

Group	Animal	Day numbers relative to Start Date: 0	21	35	70	71	72	73	74	75	76	77	78	79	80	84
2x 10^8 pfu SCV-Z/C	5548	5.7	6.0	6.3	6.6	6.8	6.5	6.6	6.7	6.7	6.7	6.7	6.7	6.6	6.7	6.7
	5545	3.1	3.1	3.3	3.3	3.5	3.3	3.4	3.4	3.4	3.4	3.3	3.3	3.4	3.5	3.4
	5546	5.6	5.8	6.0	6.3	6.3	6.2	6.2	6.2	6.2	6.3	6.3	6.2	6.2	6.5	6.3
	5555	2.4	2.4	2.6	2.6	2.6	2.6	2.6	2.5	2.5	2.5	2.6	2.5	2.6	2.7	2.7
	5558	5.7	6.1	6.1	6.2	6.1	6.0	6.1	6.1	6.0	6.1	6.0	5.9	5.8	5.9	5.8
	Mean	4.50	4.68	4.86	5.00	5.06	4.92	4.98	4.98	4.96	5.00	4.98	4.92	4.92	5.06	4.98
	S.D.	1.62	1.78	1.76	1.89	1.88	1.82	1.84	1.89	1.88	1.91	1.89	1.89	1.80	1.84	1.81
	N	5	5	5	5	5	5	5	5	5	5	5	5	5	5	5

Group	Animal	Day numbers relative to Start Date: 0	21	35	70	71	72	73	74	75	76	77	78	79	80	84
2x 10^8 pfu SCV-cont	5543	5.0	5.1	5.4	5.5	5.3	5.2	5.3	5.2	5.3	5.3	5.2	5.2	5.2	5.2	5.5
	5549	2.6	2.8	2.8	2.9	3.0	2.9	2.9	3.0	3.0	2.9	2.9	2.9	3.0	3.1	3.1
	5553	4.2	4.2	4.1	4.1	4.2	4.1	4.1	4.1	4.1	4.1	4.1	4.1	4.1	4.1	4.0
	5551	4.7	4.7	4.5	4.6	4.6	4.5	4.5	4.5	4.5	4.5	4.5	4.5	4.6	4.7	4.8
	Mean	4.13	4.20	4.20	4.28	4.28	4.18	4.20	4.20	4.23	4.20	4.18	4.18	4.23	4.28	4.35
	S.D.	1.07	1.00	1.08	1.08	0.96	0.96	1.00	0.92	0.96	1.00	0.96	0.96	0.93	0.90	1.03
	N	4	4	4	4	4	4	4	4	4	4	4	4	4	4	4

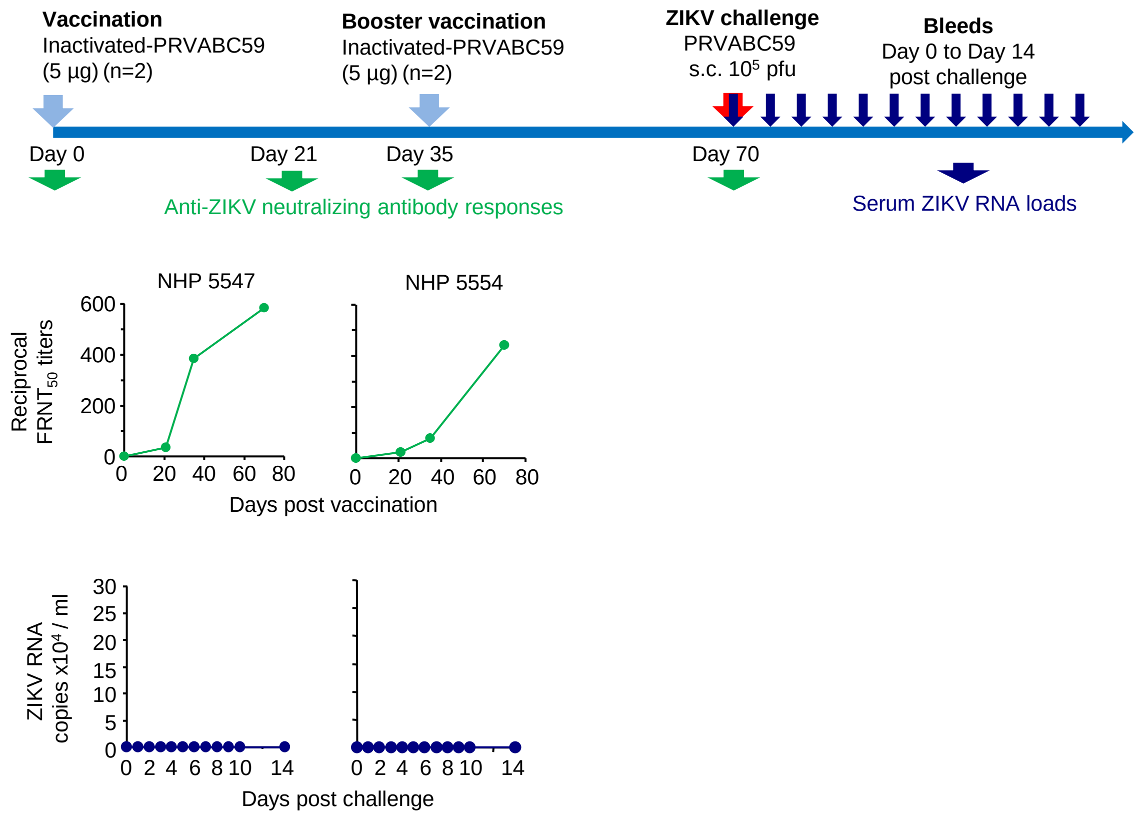

Supplementary Fig 1. Positive control ZIKV vaccine. Formalin inactivated PRVABC59 Vaccine (lot number 2016), (from Walter Reed Army Institute of Research, Springfield, MD, USA) was given twice i.m. (in 0.5 ml) as described (Rayner *et al* 2018) to 2 NHPs, with neutralizing antibody and challenge as in Fig. 1. Note vaccine and challenge strain were identical.

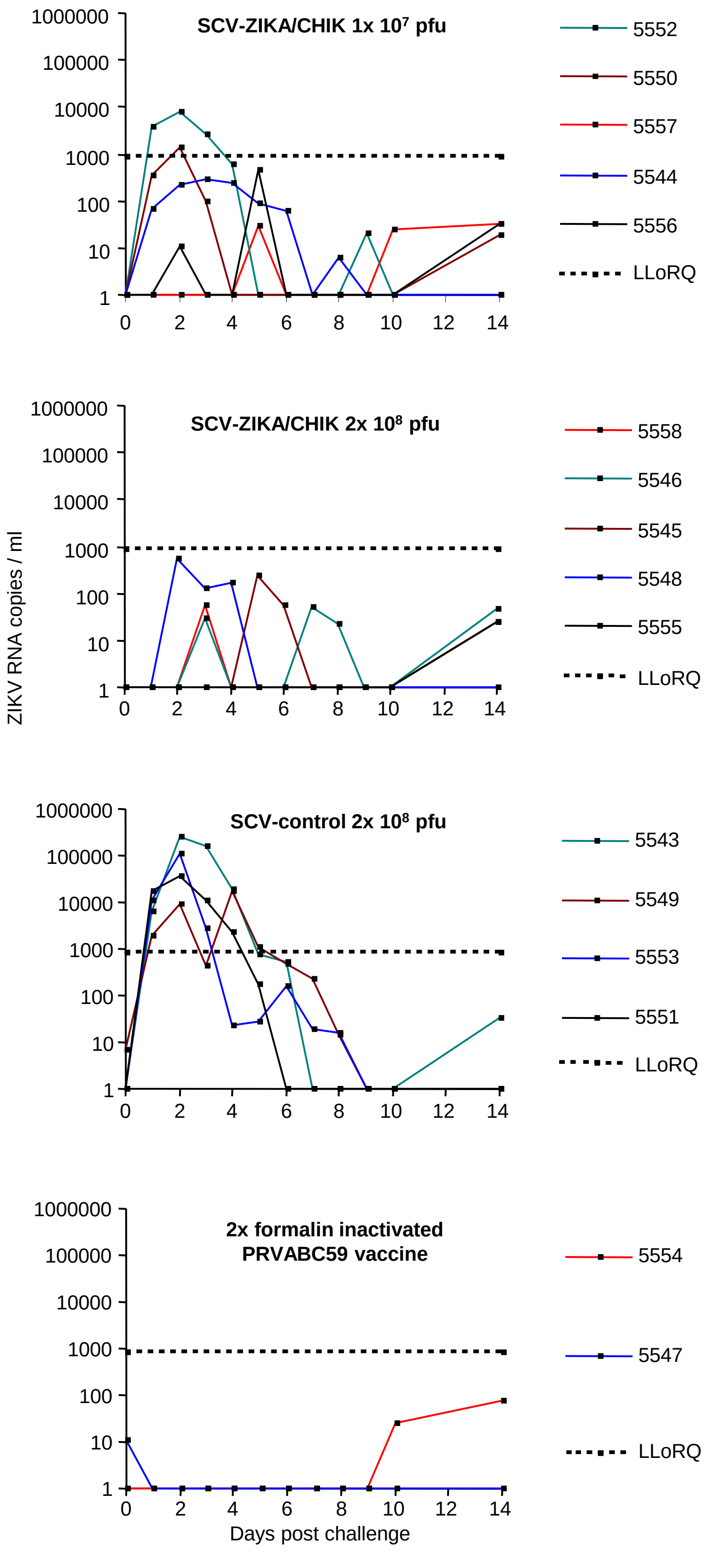

Supplementary Fig. 2. Log graphs of ZIKV RNA copies post challenge. The lower limit of reliable quantification (LLoRQ) was 860; over a large number of qRT PCR experiments using samples spiked with *in vitro*-transcribed ZIKV RNA, this was the lowest limit of 100% reliable quantification. All SCV-control vaccinated NHPs exceeded this limit on multiple days post challenge. For NHPs vaccinated once at a dose of 10^7 pfu of SCV-ZIKA/CHIK (1x 10^7), NHP 5552 exceeded this limit on 3 consecutive days clearly indicating a low level viremia. NHP 5550 also just exceeded this limit on one day, indicating a brief low level viremia. No serum samples from NHPs vaccinated twice with SCV-ZIKA/CHIK at a dose of 10^8 pfu (2x 10^8) gave qRT PCR values >860 viral RNA copy numbers per ml post-vaccination; viremia in these NHPS is thus below the limit at which virus can reliably be quantified.

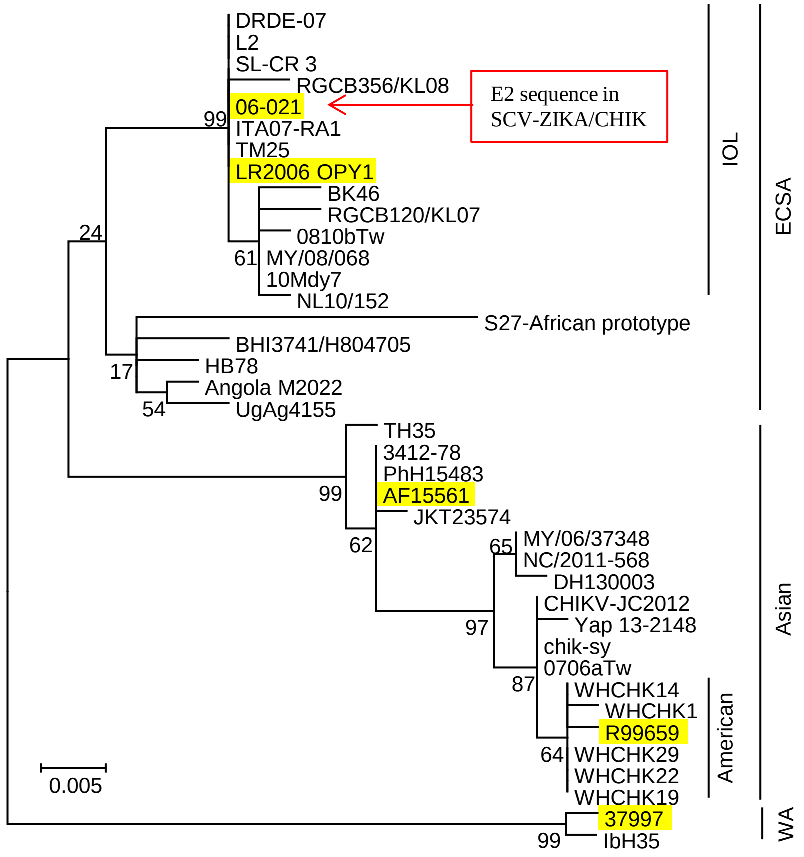

Supplementary Fig. 3. Dendogram of CHIKV E2 protein sequences showing the three genotype clusters (ECSA, Asian and WA) and the sub-lineages (IOL and American). Yellow highlights for isolates used in this study. The viruses used in the neutralization assays were: LR2006 OPY1, Tsetsarkin et al Vector Borne Zoonotic Dis. 2006, 6(4): 325-37; 37997 Vanlandingham et al Am J Trop Med Hyg. 2005, 72(5):616-21; AF15561, Harrison et al J Immunol. 1971, 107(3):643-7; and R99659, Lanciotti et al Emerg Infect Dis. 2014 20(8):1400-2.

Lineage	Strain	Accession no.	Source	Country	Year
Asian	TH35	HM045810	Human	Thailand	1958
	AF15561	EF452493	Human	Thailand	1962
	3412-78	HM045808	Human	Thailand	1978
	JKT23574	HM045791	Human	Indonesia	1983
	PhH15483	HM045790	Human	Philippines	1985
	MY/06/37348	FN295483	Human	Malaysia, Perak	2006
	0706aTw	FJ807897	Human	Indonesia (Taiwan imported case)	2007
	NC/2011-568	HE806461	Human	New Caledonia	2011
	chik-sy	KF318729	Human	China	2012
	CHIKV-JC2012	KC488650	Human	China	2012
	CHIKV-13-112A	AB860301	Human	Philippines	2013
	DH130003	KM673291	Human	Indonesia: Bali	2013
	Yap 13-2148	KJ689453	Aedes hensilli	Micronesia, Yap State	2013
	R99659	KJ451624	Human	British Virgin Islands	2014
	WHCHK29	KR559498	Human	Dominican Republic	2014
	WHCHK22	KR559491	Human	Colombia	2014
	WHCHK19	KR559488	Human	Honduras	2014
	WHCHK14	KR559483	Human	Puerto Rico	2014
	WHCHK1	KR559470	Human	Puerto Rico	2014
	WHCHK4	KR559473	Human	French Polynesia	2015
ECSA	Angola M2022	HM045823	Human	Angola	1962
	HB78	HM045822	Human	Central African Republic	1978
	UgAg4155	HM045812	Human	Uganda	1982
	L2	KF283986	Human	Comoros	2005
	06-021	AM258992	Human	Reunion	2005
	LR2006_OPY1	DQ443544	Human	Reunion	2006
	TM25	EU564334	Human	Mauritius	2006
	ITA07-RA1	EU244823	Aedes albopictus	Italy	2007
	RGCB120/KL07	GQ428213	Human	India	2007
	SL-CR 3	HM045799	Human	Sri Lanka	2007
	DRDE-07	EU372006	Human	India	2007
	0810bTw	FJ807899	Human	Malaysia	2008
	MY/08/068	FN295487	Human	Malaysia	2008
	10Mdy7	KF590564	Human	Myanmar	2010
	NL10/152	KC862329	Human	Indonesia	2010
	BK46	KJ579184	Human	Thailand	2013
	BHI3741/H804705	KP164569	Human	Brazil	2014
	S27-African prototype	AF369024	-	-	-
West African	IbH35	HM045786	Human	Nigeria	1964
	37997	AY726732	Aedes furcifer	Senegal	1983

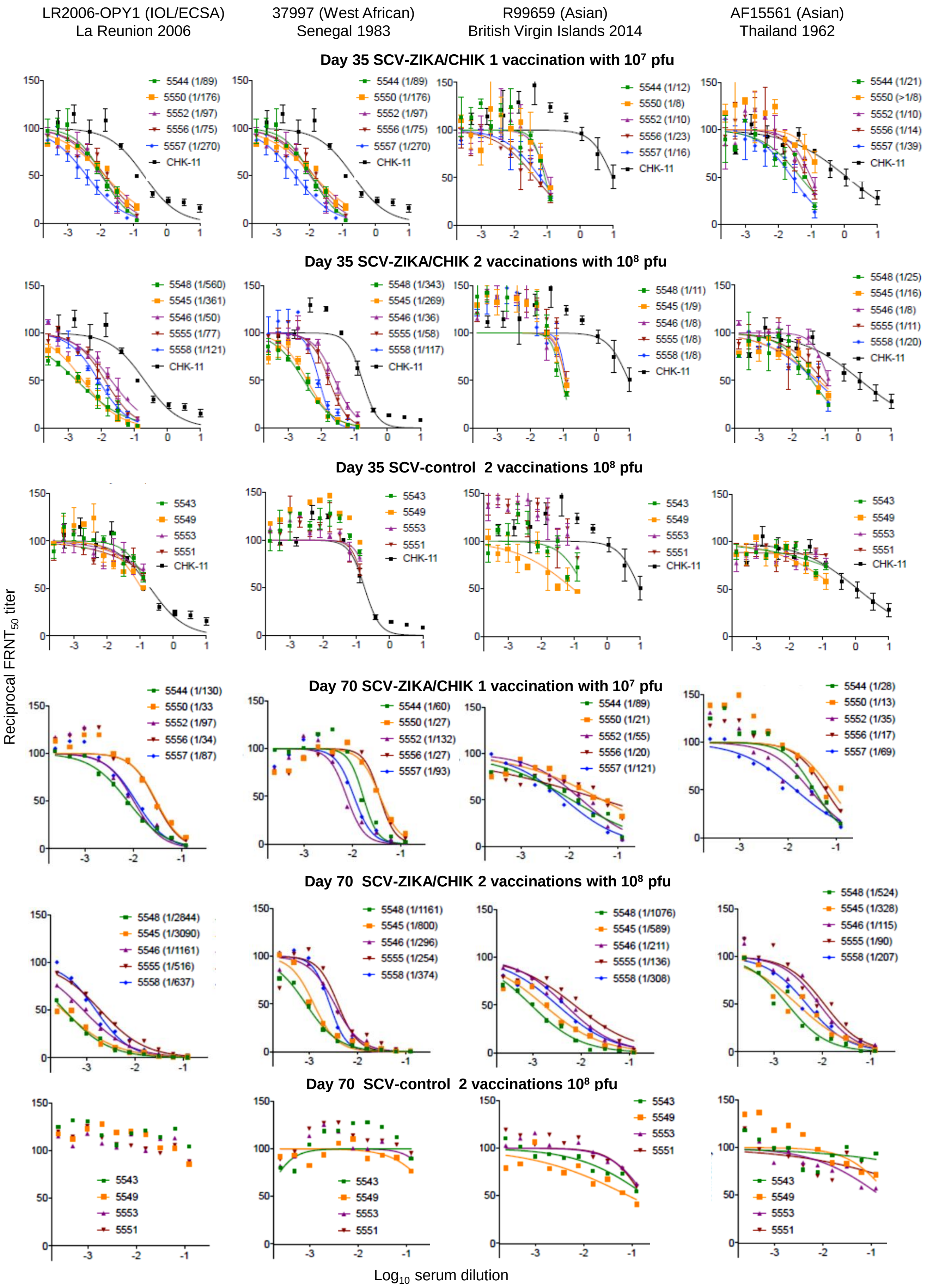

Supplementary Fig. 4. Titration curves for Fig. 3.

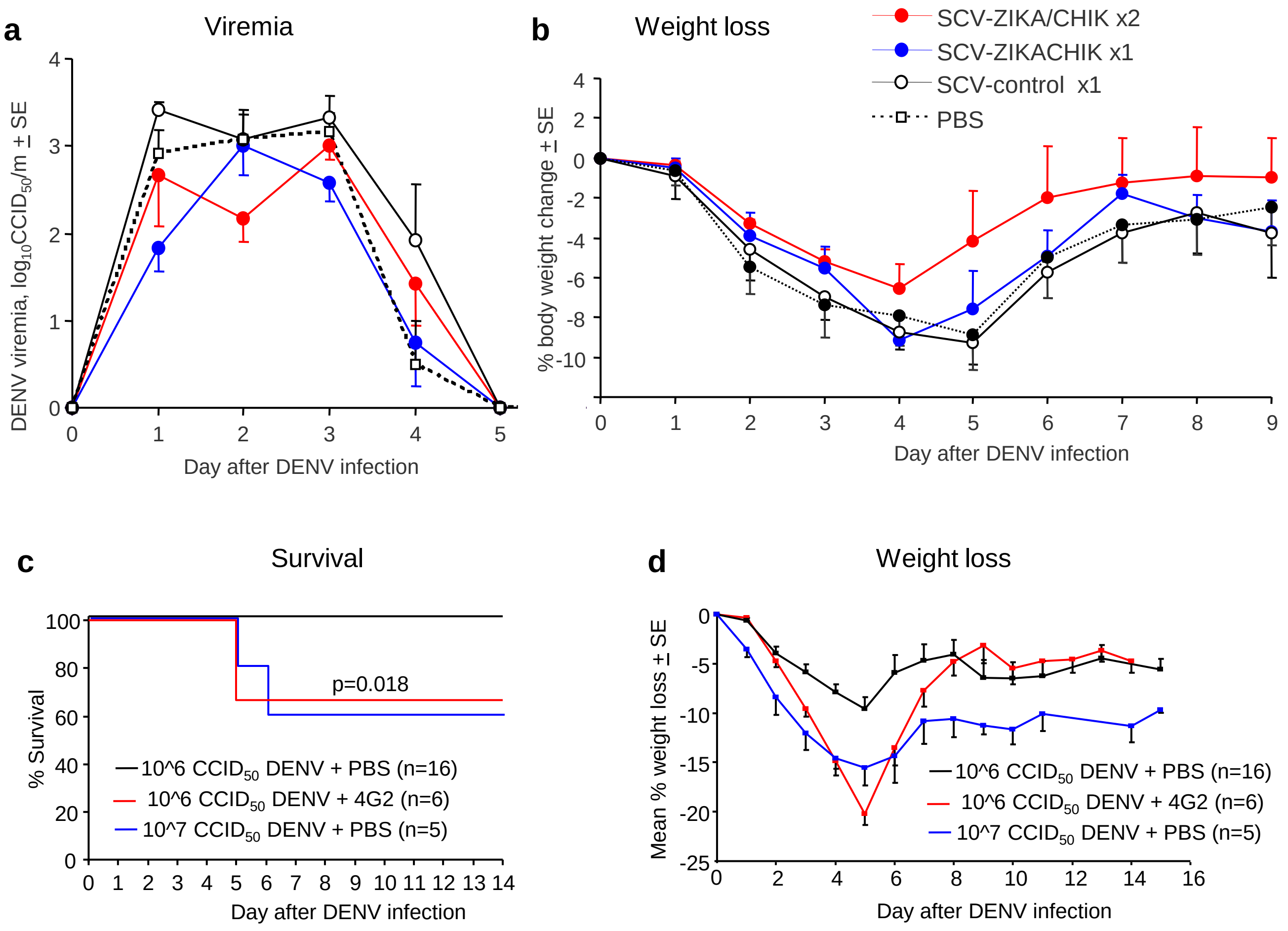

Supplementary Fig. 5. SCV-ZIKA/CHIK and DENV Antibody Dependent Enhancement (ADE). The DENV D220 mouse model in 6-10 week old female IFNAR$^{-/-}$ mice was adapted from (Sci Transl Med. 2015. 7(304):304ra142 and PLoS Pathog. 2010. 6(2):e1000790). **a** Viremia of mice infected s.c. with 10e6 $CCID_{50}$ (n=6) of DENV D220 3 weeks after vaccination with 10e6 pfu of the indicated SCV vaccines or PBS i.m. (as described Eldi et al 2017). **b** Weight loss for the same mice described in a. All mice survived, with no indication that SCV-ZIKA/CHIK vaccination exacerbated weight loss after DENV challenge.

ADE mediated by 4G4 antibody. **c** Survival of mice infected with the indicated dose of DENV D220 after receiving the anti-flavivirus antibody 4G2 i.p. (0.6 µg) or PBS, 1 day after DENV infection. Statistics by log rank (Mantel-Cox) for the10e6 dose of DENV D220 and PBS versus 4G4 antibody. **d** Weight loss for the same mice described in c. 4G4 treated mice showed significantly higher weight loss day 3 p=0.016, day 4 p=0.001, day 5 p=0.005 and day 6 p =0.007; statistics by Kolmogorov–Smirnov and t tests. Thus in this model ADE did not manifest as a change in viremia (not shown), but did increase mortality and weight loss.

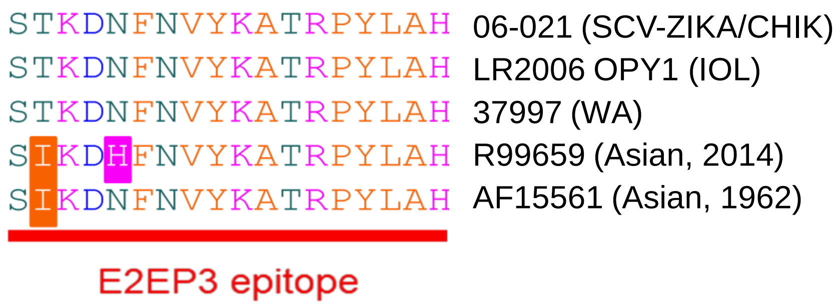

Supplementary Fig. 6. The E2EP3 epitope. In the early convalescent phase of CHIKV-infected patients and NHPs, neutralizing IgG3 responses were directed to a single linear epitope "E2EP3“, located at the N-terminus of the E2 protein. The Asian isolates have 1 and two non-conservative substitutions in this epitope.
